# Society Rights

**Published:** 1882-05

**Authors:** 


### SOCIETY RIGHTS.

The Baltimore Medical and Surgical Society recently had application from a negro doctor for membership. The applicant is said to be a well-qualified practitioner. He was promptly black-balled, and, it would seem, with some degree of enthusiasm. The *New York Medical Record* (which, by the way, makes an attenuated defense of the New York Medical Society's action in the same issue) takes the society severely to task for its action.

We may remark here that any society, medical or otherwise, has a right to control its membership. It is a question with which a non-member has nothing to do. In this case, the society exercised its own taste. We applaud its taste. That doctor (colored) has the fullest right to practice his profession. We trust he may do so successfully. But he has no right to thrust himself socially where he is not wanted. That would conflict with the rights of some one else. We do not deem it necessary to argue here that the admission of undesirable members must destroy the usefulness of any society, and often the society itself. The case is too strong to need such a forcible argument. The societies of any and all kinds in the much greater part of the United States do not care to associate with colored members. Indeed, the negro societies would rarely admit, in most sections, white members. They have the right, if it be their taste, to so exclude them. The editor of the *Record* far outstrips the civil rights bill. Even that does not undertake to control social relations. A medical society is largely a social organization. The Baltimore Medical Society has the same right to exercise its taste in the selection of its associates that the editor of the *Record* has to exercise his taste in the selection of his own.

